# A case series and literature review of immune checkpoint inhibitors-associated myocarditis (ICIM) in non-small cell lung cancer

**DOI:** 10.3389/fcvm.2025.1615085

**Published:** 2026-01-15

**Authors:** Yongzhen Sun, Yuan Liu, Yan Wang, Xiaomin Dai, Lin Shi, Jing Bi, Jing Zhang, Yuanlin Song, Jinjun Jiang, Shujing Chen

**Affiliations:** 1 Department of Respiratory and Critical Care Medicine, Affiliated Hospital of Shaoxing University, Zhejiang, China; 2 Department of Respiratory and Critical Care Medicine, Zhongshan Hospital, Fudan University, Shanghai, China; 3 Department of Cardiology, Zhongshan Hospital, Fudan University, Shanghai, China; 4 Department of Medical Oncology, Zhongshan Hospital, Fudan University, Shanghai, China; 5 Department of Rheumatology, Zhongshan Hospital, Fudan University, Shanghai, China

**Keywords:** diagnosis, immune checkpoint inhibitors, myocarditis, non-small cell lung cancer, prognosis, treatment

## Abstract

**Background:**

Immune Checkpoint Inhibitors-associated Myocarditis (ICIM) is a rare but life-threatening complication when treating Non-Small Cell Lung Cancer (NSCLC) with Immune Checkpoint Inhibitors (ICIs). This study aims to provide a foundation for optimizing the early identification, accurate stratification, and individualized treatment of ICIM.

**Methods:**

A retrospective analysis was performed on medical records of 5 NSCLC patients who developed myocarditis during ICI treatment. Data including demographics, medication history, clinical manifestations, lab tests, and imaging exams were collected, with analysis combined with a literature review.

**Results:**

The 5 patients (4 males and 1 female; aged 62–72 years, with varying NSCLC stages) developed myocardial injury within 1–3 cycles of ICI treatment. All had elevated myocardial markers and non-specific symptoms (palpitations, chest tightness, muscle weakness); 3 had abnormal electrocardiograms (ECGs). Diagnoses included 1 definite, 2 probable, and 2 possible ICIM cases. The glucocorticoid resistance rate was 80% (4/5), with only 1 patient responding effectively; mortality was 20% (1/5). No tumor progression was observed after ICI discontinuation [2 Partial Response [PR], 1 Stable Disease [SD], 1 pathological Complete Response [pCR]].

**Conclusion:**

Early identification and intervention are critical for ICIM. The core treatment is ICI discontinuation plus glucocorticoid administration, but the optimal second-line regimen for glucocorticoid-resistant patients requires further investigation.

## 1 Introduction

In recent years, immune checkpoint inhibitors (ICIs) have exhibited significant efficacy in the treatment of malignant tumors, particularly non-small cell lung cancer (NSCLC). These agents exert their effects by targeting and modulating the anti-tumor activity of T cells, inhibiting immune-suppressive pathways such as those mediated by PD-1, PD-L1, and CTLA-4. This allows T cells to function more effectively in recognizing and eliminating tumors (1, 2). However, this amplified immune response can inadvertently activate autoreactive T cells, triggering immune-related adverse events (irAEs), affecting the skin, gastrointestinal tract, endocrine system, liver, kidneys, lungs, and heart (3, 4). Among these, ICIM has become a major concern due to its high fatality rate (39.7%–50.0%), even though its overall incidence is low (0.09%–1.14%) (5–7). Fulminant myocarditis can cause cardiogenic shock or malignant arrhythmia in a short period, posing a serious threat to patients' lives. Epidemiological data shows that ICIM typically occurs 34 days after the initiation of ICIs treatment, with 81% of cases occurring within 3 months (2). In some cases, fulminant myocarditis can develop within 5 days after the first dose of medication (7), which is significantly earlier than other cardiovascular events and non-cardiac irAEs. However, there are still many controversies and areas to be explored regarding its early diagnostic methods and optimal treatment regimens.

This study retrospectively analyzed the clinical data of 5 NSCLC patients who developed myocarditis during ICIs treatment, systematically reviewed their clinical characteristics, diagnostic processes, and treatment strategies, and explored the diagnostic and therapeutic challenges and coping strategies in combination with the latest evidence-based medicine, with the goal of improving the safety of immunotherapy for NSCLC patients.

## 2 Case presentations

### 2.1 Case 1

#### 2.1.1 Baseline status

64-year-old male with a history of hypertension and diabetes mellitus (DM); Eastern Cooperative Oncology Group (ECOG) Performance Status (PS) score = 1. Tumor Diagnosis: Adenocarcinoma in the right lung upper lobe diagnosed in March 2024, stage cT2N2M1c (with pleural and bone metastases), IVB stage. Pre-treatment Tests: Cardiac biomarkers (cardiac Troponin T [cTnT], myoglobin, N-terminal pro-B-type natriuretic peptide [NT-proBNP], creatine kinase [CK], creatine kinase MB [CK-MB], etc.) were all within normal ranges; electrocardiogram (ECG) showed sinus tachycardia with occasional atrial premature beats.

#### 2.1.2 Anti-tumor treatment

From April 8 to May 22, 2024, the patient completed three cycles of chemotherapy combined with immunotherapy. Chemotherapy: Pemetrexed 980 mg (Day 1) + Carboplatin 450 mg (Day 1), every 3 weeks; Immunotherapy: Pembrolizumab 200 mg (Day 1), every 3 weeks. After 2 cycles of treatment, tumor assessment revealed partial response (PR).

#### 2.1.3 Abnormal manifestations

Nine weeks after anti-tumor treatment, the patient reported occasional palpitations, with other vital signs normal. Cardiac biomarkers showed significant elevation, including: cTnT 0.093 ng/mL (normal < 0.014 ng/mL), myoglobin 306.0 ng/mL (normal < 121.1 ng/mL), CK 509 U/L (normal 34–174 U/L), CK-MB 28 U/L (normal < 22 U/L), alanine transaminase (ALT) 65 U/L (normal 9–50 U/L), aspartate transaminase (AST) 53 U/L (normal 15–40 U/L). NT-proBNP and D-dimer levels remained within normal ranges. Electrocardiogram showed no significant changes compared with pre-treatment. Cardiac Magnetic Resonance Imaging (CMR) revealed suspected mild myocardial thickening in the lateral wall of the basal segment and suspected mild fibrosis in the same area. Coronary CT showed multiple coronary plaques with mild to moderate lumen narrowing.

#### 2.1.4 Diagnostic process

**Figure 1:**
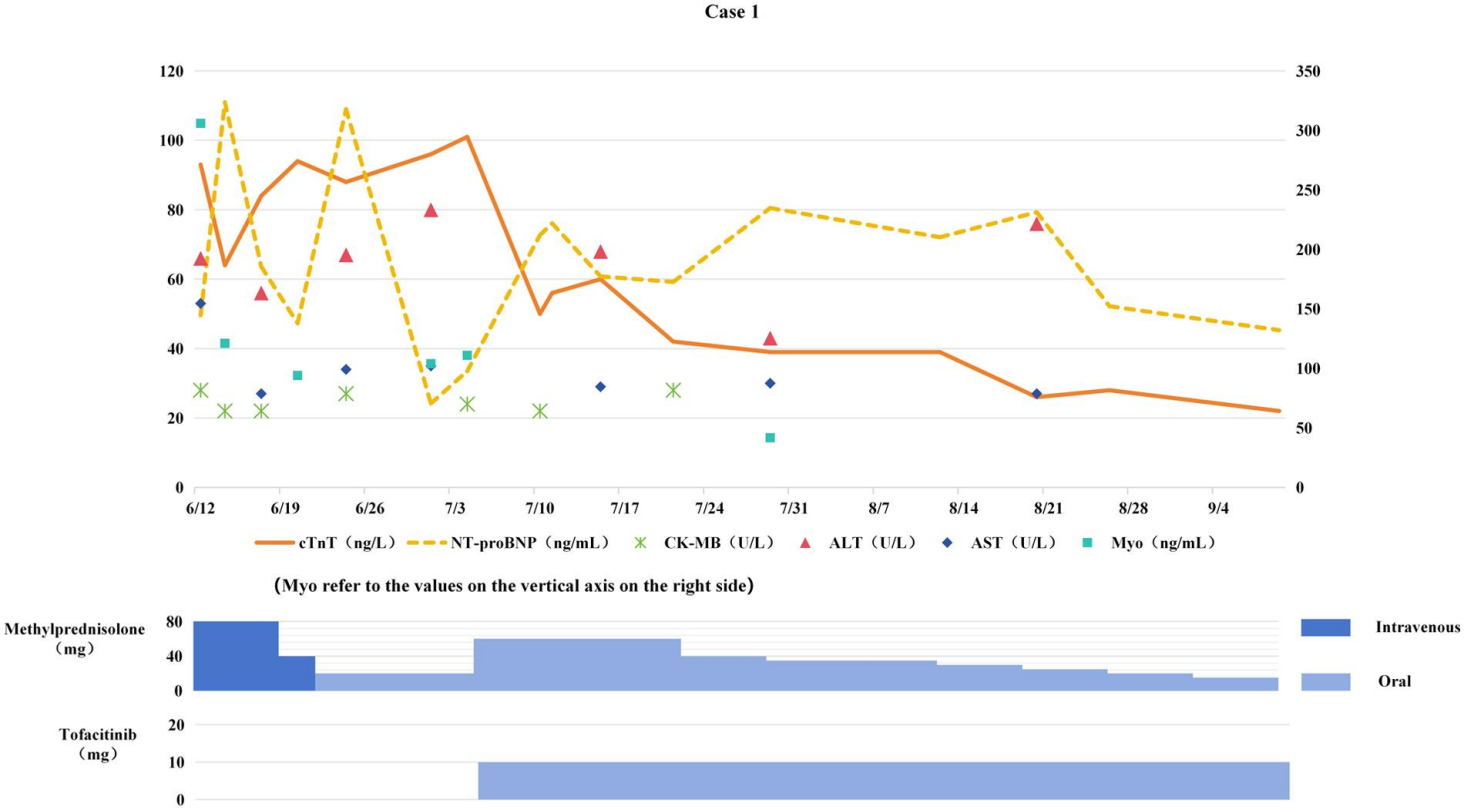
Changes in myocardial biomarkers of case 1 during treatment with glucocorticoids and tofacitinib (the x-axis represents time, and the y-axis represents the values of myocardial biomarkers. All dates are from 2024. cTnT: cardiac Troponin T, normal < 14 ng/L; Myo: Myoglobin, normal < 121.1 ng/mL; NT-proBNP: N-terminal pro-B-type natriuretic peptide, normal < 300 pg/mL; CK-MB: Creatine kinase-MB, normal < 22 U/L; ALT: Alanine transaminase, normal 9–50 U/L; AST: Aspartate transaminase, normal 15–40 U/L).

Based on the history of immunotherapy, abnormal myocardial biomarkers, contrast-enhanced cardiac MRI, and coronary CT scans, ICIM was initially considered, with coronary artery disease as a secondary consideration. Antitumor therapy was temporarily suspended. On June 12, methylprednisolone (1 mg/kg/day) was administered for anti-inflammatory treatment. However, 5 days after the initiation of steroid therapy, cTnT levels showed no improvement (see Figure 1). The next day, clopidogrel, atorvastatin, and isosorbide dinitrate were added, and the dose of methylprednisolone was gradually reduced; yet, cTnT levels continued to rise. Following a multidisciplinary team (MDT) consultation involving specialists in cardiology, pulmonology, and rheumatology, the diagnosis was subclinical ICIM (suspected) with steroid resistance.

#### 2.1.5 Treatment

On July 5, the dose of methylprednisolone tablets was increased to 60 mg daily, and tofacitinib 5 mg twice daily (BID) was added. The dose of methylprednisolone was gradually tapered, and both methylprednisolone and tofacitinib were discontinued in mid-November.

#### 2.1.6 Outcomes

Myocardial biomarkers progressively decreased to normal levels (see Figure 1). Follow-up chest CT scans on August 12 and October 14, 2024 showed the tumor remained in PR status. The original chemotherapy regimen was resumed on October 30, while immunotherapy was permanently discontinued.

### 2.2 Case 2

#### 2.2.1 Baseline status

72-year-old male with a history of hypertension; ECOG PS score = 0. Diagnosed with squamous cell carcinoma of the right lung in February 2024, stage cT1N1M0 (IIA). No abnormalities were detected in cardiac biomarkers or ECG prior to treatment.

#### 2.2.2 Anti-tumor treatment

Following multidisciplinary team consultation, preoperative neoadjuvant therapy was planned. The patient completed two cycles of chemotherapy combined with immunotherapy on March 8 and March 28, 2024. Chemotherapy regimen: Albumin-bound paclitaxel 500 mg (Day 1) + Carboplatin 400 mg (Day 1), every 3 weeks; Immunotherapy: Pembrolizumab 200 mg (Day 1), every 3 weeks. After 2 cycles of treatment chest CT showed PR.

#### 2.2.3 Abnormal manifestations

Four weeks after anti-tumor treatment, the patient developed lower limb weakness with muscle soreness. Significant elevation of cardiac biomarkers was observed on April 18, 2024, including cTnT 0.090 ng/mL, CK 6,625 U/L, CK-MB 166 U/L, myoglobin > 3,000 ng/mL, ALT 133 U/L, AST 261 U/L; NT-proBNP and D-dimer were within normal ranges. Echocardiography showed left atrial enlargement and septal thickening at the basal segment; CMR revealed suspected mild enhancement foci in the inferior septum of the left ventricle, suggesting myocarditis.

#### 2.2.4 Diagnostic process

Following MDT discussions, the diagnosis of immune-mediated myositis complicated with ICIM was confirmed based on the history of immunotherapy, myalgia symptoms, significantly elevated cardiac biomarkers, and cardiac MRI findings.

#### 2.2.5 Treatment

Immunotherapy was discontinued. Starting from April 22, methylprednisolone pulse therapy (500 mg daily for 3 consecutive days) was administered along with intravenous immunoglobulin (IVIG) (total dose 2 g/kg) for immune modulation. After one week of treatment, persistent elevation of cTnT levels suggested steroid resistance. Oral mycophenolate mofetil (0.75 g twice daily) was added, followed by IVIG 20 g once daily for three consecutive days. Trimethoprim-sulfamethoxazole (TMP-SMX) (160/800 mg/day) was also administered for Pneumocystis jirovecii pneumonia (PCP) prophylaxis.

#### 2.2.6 Outcomes

**Figure 2:**
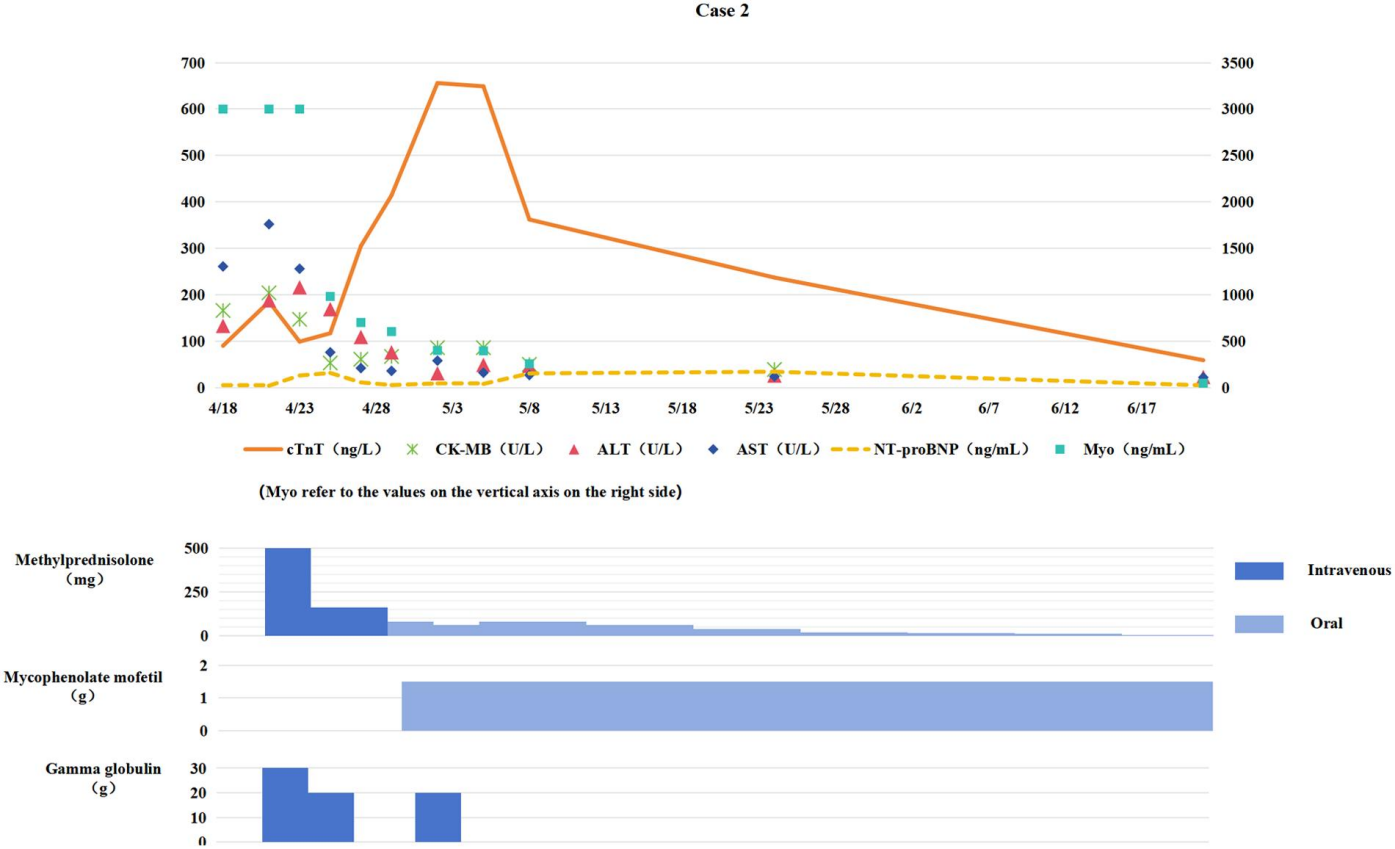
Changes in myocardial biomarkers of case 2 during treatment with glucocorticoids, mycophenolate mofetil and gamma globulin (the x-axis represents time, and the y-axis represents the values of myocardial markers. All dates are from 2024. cTnT: cardiac Troponin T, normal <14 ng/L; Myo: Myoglobin, normal <121. 1 ng/mL; NT-proBNP: N-terminal pro-B-type natriuretic peptide, normal <300 pg/mL; CK-MB: Creatine kinase myocardial band, normal <22 U/L; ALT: Alanine transaminase, normal 9–50 U/L; AST: Aspartate transaminase, normal 15–40 U/L).

Cardiac biomarkers gradually decreased (see Figure 2), with significant improvement in fatigue and myalgia. Following 4 weeks of anti-ICIM treatment, a follow-up chest CT scan showed persistent PR status. The chemotherapy regimen was resumed the next day, while immunotherapy was permanently discontinued. Corticosteroids were tapered and discontinued one month later, and the patient subsequently underwent radical lung cancer resection at the Department of Thoracic Surgery on the following day. Postoperative pathological examination revealed pathological Complete Response (pCR).

### 2.3 Case 3

#### 2.3.1 Baseline status

62-year-old male with a history of DM; ECOG PS score = 0. Diagnosed with squamous cell carcinoma of the right upper lobe of the lung in April 2024, stage cT4N2M0 (IIIB). Pre-treatment evaluations: ECG showed sinus tachycardia; cardiac biomarkers: cTnT 0.015 ng/mL, NT-proBNP 464 pg/mL, while CK, CK-MB, ALT, and AST levels were normal; echocardiography revealed left atrial enlargement.

#### 2.3.2 Anti-tumor treatment

The patient completed three cycles of chemotherapy combined with immunotherapy from April 25 to June 6, 2024, as follows: Chemotherapy regimen: Albumin-bound paclitaxel 500 mg (Day 1) + Carboplatin 550 mg (Day 1), every 3 weeks; Immunotherapy: Tislelizumab 200 mg (Day 1), every 3 weeks. After 2 cycles of treatment, chest CT assessment revealed PR.

#### 2.3.3 Abnormal manifestations

Eight weeks after anti-tumor treatment, the patient developed chest tightness and generalized fatigue. Emergency examination on June 28 showed significantly elevated cardiac biomarkers: cTnT 2.890 ng/mL, CK 6,863 U/L, CK-MB 189 U/L, NT-proBNP 2,435 pg/mL (normal <300 pg/mL), ALT 290 U/L, AST 303 U/L, D-dimer 0.32 mg/L (normal <0.5 mg/L). ECG revealed atrial fibrillation with ST-segment elevation. Echocardiography showed reduced contractile activity in the anterior wall of the left ventricle and a left ventricular ejection fraction (LVEF) of 56%. Subsequent coronary CT angiography (CTA) demonstrated multiple coronary plaques [moderate-to-severe stenosis in the proximal segment of the left anterior descending artery (LAD), with mild to moderate stenosis in other segments]. CMR revealed mild global left ventricular systolic dysfunction, bilateral atrial enlargement, and right ventricular enlargement with an LVEF of 49%.

#### 2.3.4 Diagnostic process

After cardiology consultation, myocardial injury [acute coronary syndrome (ACS)? ICIM?] was considered. The patient and his family refused emergency coronary angiography. Following antiplatelet therapy, anticoagulation, and coronary vasodilation treatment, myocardial biomarkers did not decrease, leading to a strong suspicion of ICIM.

#### 2.3.5 Treatment

On June 28, methylprednisolone was added at 2 mg/kg/day for anti-inflammatory therapy, combined with IVIG (total dose 2 g/kg) for immune modulation. Improvement in symptoms led to switching to oral prednisone for sequential therapy.

#### 2.3.6 Outcomes

**Figure 3:**
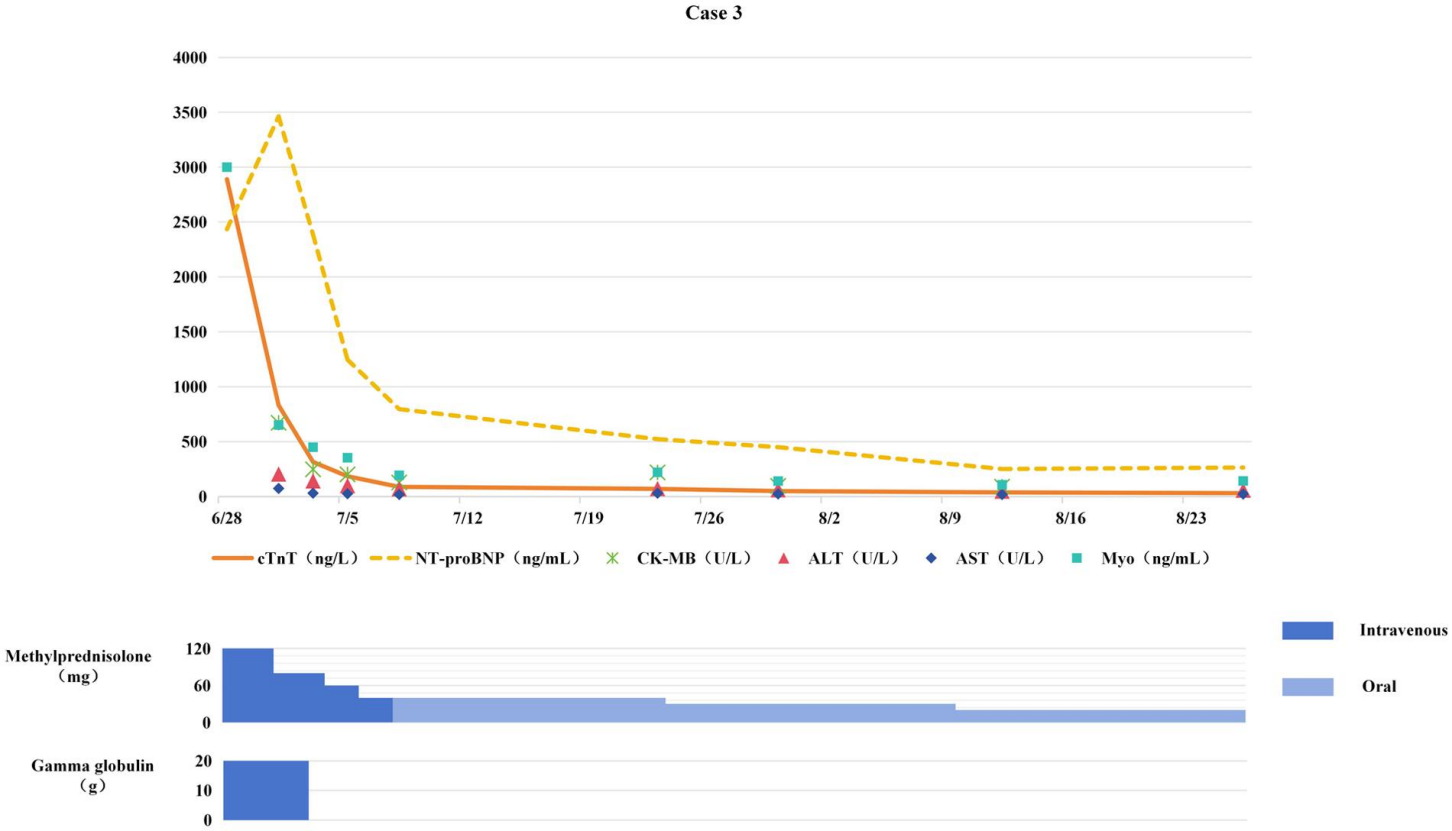
Changes in myocardial biomarkers of case 3 during treatment with glucocorticoids and gamma globulin (the x-axis represents time, and the y-axis represents the values of myocardial markers. All dates are from 2024. cTnT: cardiac Troponin T, normal <14 ng/L; Myo: Myoglobin, normal <121. 1 ng/mL; NT-proBNP: N-terminal pro-B-type natriuretic peptide, normal <300 pg/mL; CK-MB: Creatine kinase myocardial band, normal <22 U/L; ALT: Alanine transaminase, normal 9–50 U/L; AST: Aspartate transaminase, normal 15–40 U/L).

Chest tightness and fatigue improved; myocardial biomarkers gradually decreased toward normal levels (see Figure 3); ECG restored sinus rhythm with ST-segment normalization. Chemotherapy was resumed after 4 weeks of anti-ICIM treatment, and immunotherapy was permanently discontinued. Subsequent chest CT revealed no tumor progression, with the patient maintaining PR. The patient completed 6 cycles of chemotherapy followed by sequential radiotherapy on December 23, 2024. After chemotherapy and radiotherapy, the patient remained stable without significant abnormalities.

### 2.4 Case 4

#### 2.4.1 Baseline status

67-year-old female with no comorbidities; ECOG PS score = 1. Diagnosed with adenocarcinoma of the right upper lobe of the lung in June 2024, stage cT4N3M0 (III-C). Pre-treatment evaluations: ECG and cardiac biomarkers were within normal ranges.

#### 2.4.2 Anti-tumor treatment

The patient completed two cycles of chemotherapy combined with immunotherapy on June 21 and July 12, 2024. Chemotherapy regimen: Pemetrexed 740 mg (Day 1) + Carboplatin 380 mg (Day 1), every 3 weeks; Immunotherapy: Tislelizumab 200 mg (Day 1), every 3 weeks.

#### 2.4.3 Abnormal manifestations

Four weeks after anti-tumor treatment, the patient developed fatigue, bilateral ptosis, and blurred vision. Follow-up on August 1 showed elevated cardiac biomarkers: cTnT 0.195 ng/mL, CK 3,989 U/L, CK-MB 119 U/L, ALT 116 U/L, AST 208 U/L; NT-proBNP was normal. ECG showed ST-segment depression; Enhanced MRI revealed slightly uneven signals in the extraocular muscles; Coronary CTA and cardiac CMR showed no significant abnormalities.

#### 2.4.4 Diagnostic process

Based on the presence of ptosis, elevated cardiac biomarkers, and abnormal ECG findings following immunotherapy, a diagnosis of possible ICIM with concomitant myositis was established.

#### 2.4.5 Treatment

**Figure 4:**
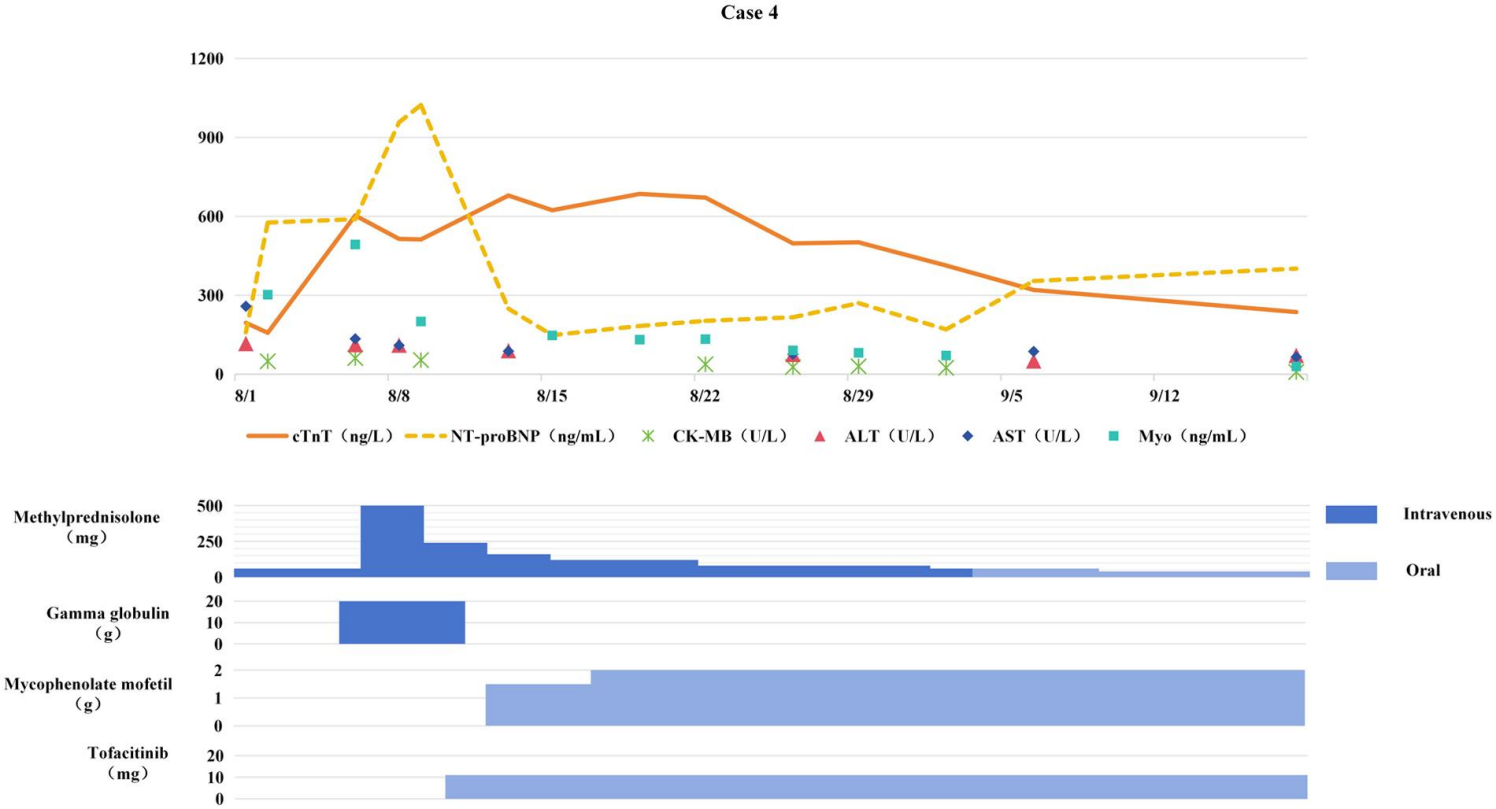
Changes in myocardial biomarkers of case 4 during treatment with glucocorticoids, gamma globulin, mycophenolate mofetil and tofacitinib (the x-axis represents time, and the y-axis represents the values of myocardial markers. All dates are from 2024. cTnT: cardiac Troponin T, normal <14 ng/L; Myo: Myoglobin, normal <121. 1 ng/mL; NT-proBNP: N-terminal pro-B-type natriuretic peptide, normal <300 pg/mL; CK-MB: Creatine kinase myocardial band, normal <22 U/L; ALT: Alanine transaminase, normal 9–50 U/L; AST: Aspartate transaminase, normal 15–40 U/L).

Consequently, immunotherapy was discontinued and methylprednisolone therapy (1 mg/kg/day) was initiated. One week later, serum cTnT levels continued to rise (see Figure 4). Methylprednisolone pulse therapy (500 mg/day for 3 days) combined with IVIG (2 g/kg) was administered, but cTnT remained elevated, confirming steroid-resistant ICIM. Subsequently, tofacitinib (11 mg/day) was added, followed by mycophenolate mofetil (0.75 g BID).

#### 2.4.6 Outcomes

The addition of tofacitinib and mycophenolate mofetil significantly improved symptoms such as ptosis, with cTnT gradually decreasing. After 3 months of anti-ICIM treatment, the patient developed chest tightness and dyspnea. Chest CT revealed newly developed multiple pulmonary inflammatory lesions, and the patient was confirmed to have PCP by etiological examination. Mycophenolate mofetil and tofacitinib were discontinued, and anti-PCP treatment was initiated. Pulmonary inflammation gradually subsided, but myocardial markers elevated again. After pneumonia control, mycophenolate mofetil was resumed at 0.5 g BID, resulting in a gradual decrease in myocardial markers. After discontinuing anti-tumor therapy for 4 months, follow-up chest CT showed stable disease (SD). Since then, the patient and her family refused anti-tumor treatment, and tumor progression occurred 5 months later.

### 2.5 Case 5

#### 2.5.1 Baseline status

A 64-year-old male with no clear underlying diseases; ECOG PS score = 1. Diagnosed with lung adenocarcinoma at an external hospital in June 2024, staged cT2aN3M1b (with adrenal gland metastasis), stage IVA. Pre-treatment auxiliary examinations indicated that myocardial biomarkers and ECG were normal.

#### 2.5.2 Anti-tumor treatment

The patient previously received a chemotherapy regimen (pemetrexed 800 mg on Day 1 + carboplatin 450 mg on Day 1, once every 3 weeks) combined with bevacizumab (700 mg on Day 1, once every 3 weeks) for anti-angiogenic targeted therapy. Due to severe myelosuppression, the regimen was changed on August 27 to pembrolizumab immunotherapy (200 mg, once every 3 weeks).

#### 2.5.3 Abnormal manifestations

Six weeks after ICI initiation, the patient was admitted to the emergency department due to chest tightness, shortness of breath, and fever (maximum body temperature 39 °C). Emergency Examination Results: Procalcitonin (PCT) 3.83 ng/mL, cTnT 0.041 ng/mL, NT-proBNP 4,362 pg/mL, D-dimer 6.83 mg/L, hemoglobin (Hb) 75 g/L, platelets (PLT) 75 × 10⁹/L, white blood cells (WBC) 3.84 × 10⁹/L, C-reactive protein (CRP) 90.0 mg/L; ECG showed sinus tachycardia, which later converted to atrial fibrillation; echocardiography indicated enlargement of both atria, moderate-to-severe mitral regurgitation (MR), and severe tricuspid regurgitation (TR). Chest CT revealed a right lung mass, bilateral pulmonary exudative lesions, and a small amount of bilateral pleural effusion; results of peripheral blood metagenomic next-generation sequencing (mNGS, including both DNA and RNA) and blood culture were all negative.

#### 2.5.4 Diagnostic and treatment process

**Figure 5:**
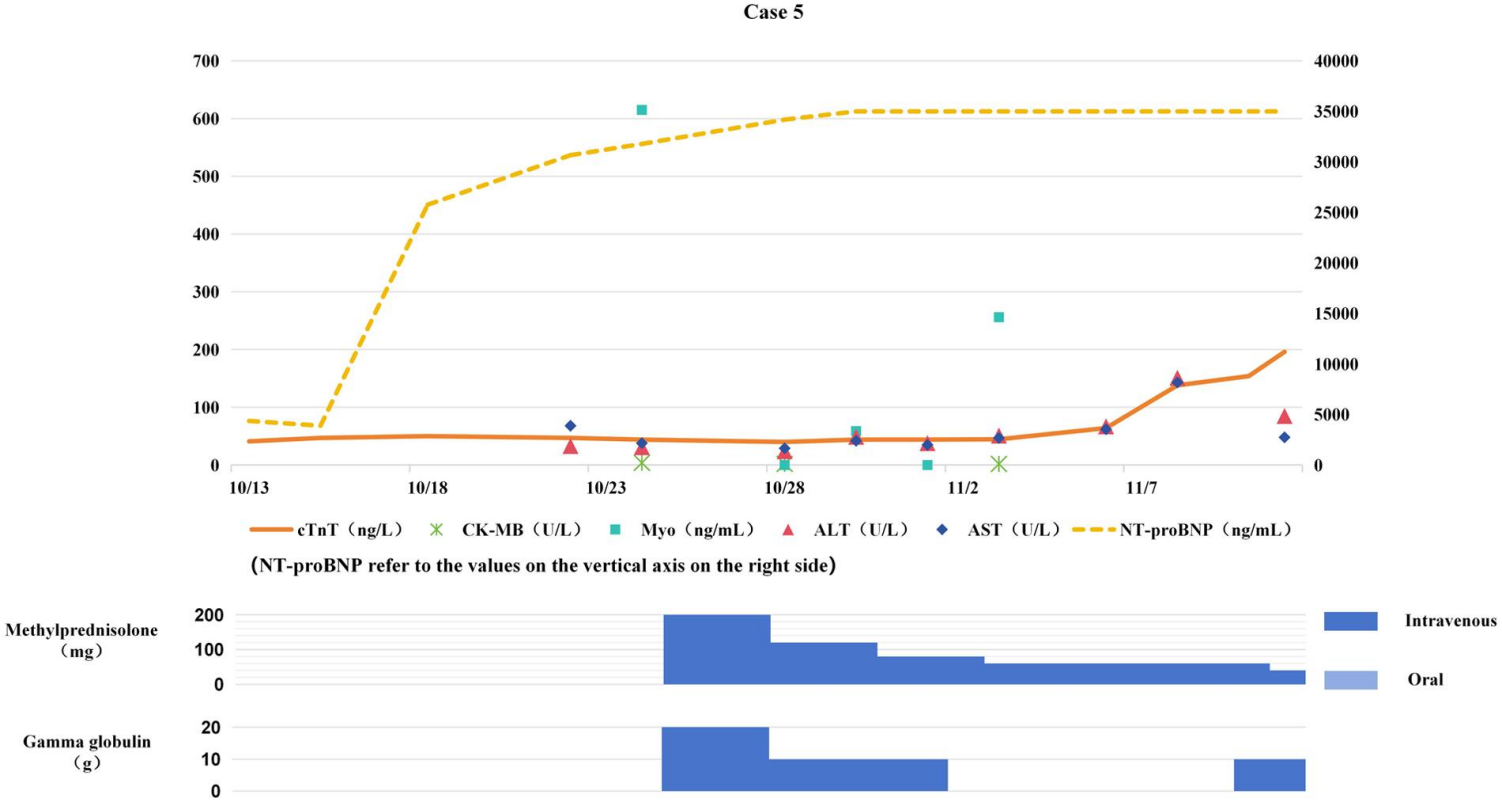
Changes in myocardial bimarkers of case 5 during treatment with glucocorticoids and gamma globulin (the x-axis represents time, and the y-axis represents the values of myocardial markers. All dates are from 2024. cTnT: cardiac Troponin T, normal <14 ng/L; Myo: Myoglobin, normal <121. 1 ng/mL; NT-proBNP: N-terminal pro-B-type natriuretic peptide, normal <300 pg/mL; CK-MB: Creatine kinase myocardial band, normal <22 U/L; ALT: Alanine transaminase, normal 9–50 U/L; AST: Aspartate transaminase, normal 15–40 U/L).

Based on clinical manifestations and examination findings, the patient was diagnosed with severe pneumonia, septic shock, and heart failure. Norepinephrine was administered to maintain blood pressure, meropenem for anti-infective therapy, and bilevel positive airway pressure (BiPAP) for respiratory support, along with diuretic therapy and myocardial nutritional support. Due to severe respiratory failure, endotracheal intubation was performed one week later. On treatment day 15, PCT was 0.42 ng/mL, serum creatinine (Scr) 173 μmol/L, WBC count 7.9 × 10⁹/L, and CRP 21.8 mg/L. The patient's body temperature decreased, but myocardial markers continued to rise (see Figure 5). Considering the patient's history of immunotherapy, cardiology consultation suggested possible ICIM. Consequently, intravenous methylprednisolone 4 mg/kg daily was initiated, combined with IVIG (total dose 2 g/kg) for immune modulation. On hospital day 15, the patient developed significant bradycardia and underwent temporary cardiac pacemaker implantation. On hospital day 17, ventricular tachycardia occurred and was treated with lidocaine and amiodarone. On hospital day 19, ventricular fibrillation occurred, which was successfully converted with electrical defibrillation. Reevaluation at this time showed: cTnT 0.047 ng/mL, NT-proBNP 30,664.0 pg/mL, PCT 0.81 ng/mL, WBC count 7.88 × 10⁹/mL, and CRP 31.1 mg/mL. Repeat echocardiography indicated biatrial enlargement, moderate-to-severe mitral regurgitation, severe tricuspid regurgitation, and a small amount of pericardial effusion, with no evidence of valvular vegetations. Repeated electrocardiograms showed no dynamic ST-T segment changes. Cardiology was consulted again, and given the diagnosis of fulminant ICIM, additional intravenous IVIG therapy was administered.

#### 2.5.5 Outcomes

During treatment, cTnT and NT-proBNP continued to rise (see Figure 5), and malignant arrhythmias occurred repeatedly. On hospital day 21, ventricular fibrillation occurred again, and the patient died despite aggressive resuscitative efforts.

## 3 Literature review and discussion

We conducted a retrospective analysis of the clinical characteristics (see Table 1) and treatment outcomes of ICIM in five patients with NSCLC who had received ICIs. Our findings highlight the complexity of ICIM and the challenges involved in its diagnosis and treatment. We systematically examined the challenges in diagnosing and treating ICIM, as well as the factors influencing prognosis, based on the latest guidelines and evidence-based data. Our aim is to provide valuable insights for optimizing clinical practice.

**Table 1:** Basic clinical characteristics of 5 patients with ICI-associated myocarditis.

Clinical characteristic	Case 1	Case 2	Case 3	Case 4	Case 5
Gender	Male	Male	Male	Female	Male
Age (years)	64	72	62	67	64
NSCLC stage	Stage ⅣB	Stage ⅡA	Stage ⅢB	Stage ⅢC	Stage ⅣA
ICI	Pembrolizumab	Pembrolizumab	Tislelizumab	Tislelizumab	Pembrolizumab
Underlying diseases	Hypertension, Type 2 DM	Hypertension	Type 2 DM	None	None
Cycles of immunotherapy	3	2	3	2	1
Clinical manifestations	Occasional palpitations	Fatigue, bilateral lower limb muscle soreness	Chest tightness, general fatigue	Fatigue, ptosis, blurred vision	Chest tightness, shortness of breath

NSCLC, non-small cell lung cancer; ICI, immune checkpoint inhibitor; DM, diabetes mellitus.

Literature Review Methods: We searched the PubMed database from its inception to December 31, 2024, to include the latest research on the diagnosis, treatment, and prognosis of ICIM. The detailed search strategy for PubMed is provided as an example: (“Immune Checkpoint Inhibitor-induced Myocarditis” OR “ICIM” OR “Immune Checkpoint Inhibitor Cardiotoxicity” OR “ICI-associated Myocarditis”) AND (“Non-Small Cell Lung Cancer” OR “NSCLC”) AND (“Diagnosis” OR “Treatment” OR “Prognosis” OR “Steroid Resistance” OR “Fulminant Myocarditis”).

### 3.1 Diagnosis of ICIM

The diagnosis of ICIM is currently based on a combination of clinical manifestations, myocardial biomarkers, electrocardiography (ECG), and imaging studies. However, these diagnostic indicators have limitations, and the academic community is working to improve diagnostic accuracy by optimizing diagnostic criteria. The 2022 Guidelines of the European Society of Cardiology (ESC) recommend a “1 major+2 minor” criterion-based approach for diagnosing ICIM. This approach highlights the importance of using cardiac MRI (following the Lake Louise criteria) for early detection (8). Additionally, the Bonaca stratification criteria use a three-level classification of “definite, probable, and possible” to avoid excluding potential ICIM cases (9). In this study, the Bonaca diagnostic criteria were used to diagnose and stratify the five patients, with one definite, two probable, and two possible cases. According to the 2022 ESC guidelines, the patients were further classified as either hormone-sensitive (one case) or hormone-resistant (four cases). The severity of ICIM was graded according to the guidelines of the American Society of Clinical Oncology (ASCO) (10): one case was classified as grade G1, one as G2, two as G3, and one as G4 (Table 2).

**Table 2:** Diagnostic findings and stratification of 5 patients.

Diagnostic category	Case 1	Case 2	Case 3	Case 4	Case 5
ECG					
ST segment changes	None reported	None reported	Elevation (≤5 mm in leads Ⅰ, aVL, Ⅱ, V1-V6)	Horizontal depression (<1 mm in leads Ⅰ, aVL, V2-V5)	None reported
Arrhythmias	Sinus tachycardia	Sinus rhythm (no arrhythmia)	Atrial fibrillation	None reported	Sinus tachycardia/bradycardia; atrial fibrillation/flutter; ventricular tachycardia/fibrillation
Echocardiogram					
Cardiac structure changes	Left atrial enlargement; ascending aortic dilatation	Left atrial enlargement; ascending aortic dilatation	Bilateral atrial enlargement; right ventricular enlargement	No abnormalities	Bilateral atrial enlargement; small pericardial effusion
Valvular findings	Aortic valve calcification	No abnormalities	No abnormalities	No abnormalities	Moderate-severe mitral regurgitation (MR); severe tricuspid regurgitation (TR); mild aortic regurgitation (AR)
Ventricular wall motion	No abnormalities reported	Basal interventricular septal thickening	Weakened systolic motion of left ventricular anterior wall	No abnormalities	No abnormalities reported
LVEF (%)	71	63	56	No abnormalities	61
CMR					
Late gadolinium enhancement (LGE)	Suspected minimal enhancement (basal lateral wall)	Suspected minimal enhancement (left ventricular inferior septum)	Not specified	No LGE reported	Not performed
Other findings	Suspected minimal fibrosis (basal lateral wall); left atrial enlargement; LVEF = 52%	Basal interventricular septal thickening; mild aortic regurgitation; LVEF = 60.4%	Mild left ventricular systolic dysfunction; bilateral atrial+right ventricular enlargement; LVEF = 49%	Mild mitral/aortic regurgitation; minimal pericardial effusion; LVEF = 66%	Not performed
Coronary CTA	LAD: 50–60%, RCA: 55–65%; unstable plaque (proximal LAD)	Not performed	proximal LAD: 65–70%	Myocardial bridge (proximal-middle LAD)	Not performed
Bonaca diagnostic stratification	Probable diagnosis	Definitive diagnosis	Probable diagnosis	Possible diagnosis	Possible diagnosis
ASCO Severity grade	G1	G2	G3	G3	G4
Glucocorticoid response	Resistant	Resistant	Sensitive	Resistant	Resistant

ECG, electrocardiogram; LVEF, left ventricular ejection fraction; CMR, cardiac magnetic resonance; CTA, computed tomography angiography; LAD, left anterior descending artery; RCA, right coronary artery; ASCO, American Society of Clinical Oncology.

#### 3.1.1 Clinical manifestations

The symptoms of ICIM are diverse and may include chest pain, palpitations, dyspnea, fatigue, dizziness, and syncope. These symptoms may be masked by or coexist with other irAEs or pulmonary symptoms related to the underlying malignancy and comorbid conditions. Approximately 20% to 30% of patients with ICIM also exhibit manifestations of myositis or muscle weakness (6, 11), suggesting immune cross-reactions. The underlying mechanism may be related to the shared antigenicity of α-myosin in both cardiac and skeletal muscles (12). Among the five cases, in addition to cardiovascular symptoms, Case 2 also experienced limb soreness, while Case 4 presented with muscle weakness, including fatigue and eyelid ptosis (Table 1).

#### 3.1.2 Myocardial markers

In the early stages of ICIM, some patients may have subtle symptoms, and the only indication of myocardial injury may be an increase in certain biomarkers. Among these markers, cTnT is the most important for diagnosing ICIM. According to prospective studies, cTnT has the highest positive rate (98%) within 72 h, which is significantly higher than that of cTnI (88%) and CK (75%) (13). Additionally, BNP can assist in the diagnosis of ICIM, with a positive rate of 66% (7). It can be used as a supplementary indicator to assess cardiac function. In addition to these myocardial markers, biomarkers related to the liver and skeletal muscles also have diagnostic value for ICIM. Studies have shown that at least three of the following biomarkers—AST, ALT, CK, and lactate dehydrogenase (LDH)—are elevated in 95% of ICIM patients (14).

In the five cases reported in this study, all patients showed varying degrees of cTnT elevation, accompanied by elevations in NT-proBNP (3/5), CK-MB (4/5), myoglobin (5/5), ALT (5/5), and AST (5/5). Therefore, when suspecting ICIM, monitoring cTnT and assessing changes in liver enzymes and skeletal muscle biomarkers are essential.

#### 3.1.3 ECG

ECG abnormalities in patients with ICIM are highly diverse and may include supraventricular arrhythmias, ventricular arrhythmias, heart block, as well as new ST-T segment changes and T-wave abnormalities unrelated to coronary artery disease (15). Complete heart block, ventricular arrhythmias, and pathological Q-waves are associated with an increased 30-day all-cause mortality (16). However, the ECGs of some patients may show no abnormalities (16, 17).

In three out of the five cases in this study, different ECG changes were observed (see Table 2). Although these ECG changes are not specific, they still have certain auxiliary value for the early identification of ICIM and can provide valuable time for subsequent treatment. It is worth noting that in the early stages, the ECGs of some ICIM patients may be normal or show only mild abnormalities. As inflammation spreads from focal to diffuse, electrocardiogram conduction abnormalities can progress significantly within a short time (18). For example, in Case 5, the early-stage ECG only indicated sinus tachycardia, but it progressed to malignant arrhythmia rapidly, ultimately leading to the patient's demise.

#### 3.1.4 Echocardiogram

Echocardiography is a valuable tool for evaluating changes in cardiac structure and function, such as reduced LVEF and abnormal myocardial motion. However, in some cases, even with normal LVEF, global longitudinal strain (GLS) may decrease (19). Notably, these changes may not be significant in the early stages of myocarditis, making it difficult to confirm the diagnosis using echocardiography alone.

Cardiac structural abnormalities were identified in 4 of the 5 cases in this cohort (Table 2), as follows: atrial and/or ventricular chamber enlargement in all 4 cases; interventricular septal thickening in 1 case; severe valvular regurgitation in 1 case. Notably, despite these structural alterations, none of the patients exhibited a clinically significant decline in LVEF.

#### 3.1.5 Cardiac magnetic resonance imaging (CMR) examination

According to the 2018 Lake Louise criteria (20), CMR is a core tool for the diagnosis of myocarditis, with high accuracy. It not only clearly demonstrates the typical features of myocardial inflammation, edema, and fibrosis but also identifies the specific location, extent, and severity of myocardial inflammation. However, it has clinical limitations: in the early disease stage, myocardial inflammation may not induce obvious CMR signal changes, and the prolonged examination duration poses risks for critically ill patients.

Among the five clinical cases summarized in Table 2, distinct cardiovascular manifestations were observed: Two cases exhibited suspected myocardial late gadolinium enhancement (LGE) on CMR imaging; one case presented both LGE and interventricular septal thickening; one case demonstrated reduced left ventricular systolic function with decreased EF; one case showed no significant structural or functional abnormalities; the remaining case was excluded from imaging analysis due to critical clinical instability. Notably, discrepancies in LVEF quantification were identified between CMR and echocardiography. Suboptimal acoustic windows during echocardiographic examination may lead to underestimation of left ventricular volume measurements, whereas CMR's three-dimensional reconstruction capability provides superior spatial resolution and volumetric accuracy, thereby serving as the gold standard for LVEF assessment. Nevertheless, echocardiography remains a pragmatic alternative for longitudinal monitoring of cardiac function in clinical practice, owing to its accessibility and non-invasiveness.

#### 3.1.6 Myocardial biopsy

Myocardial biopsy is the gold standard for diagnosing myocarditis (10, 21). However, due to its invasive nature, it carries certain risks, such as bleeding and arrhythmia, which limit its widespread clinical practice. Given the low acceptance of myocardial biopsy among patients and the associated procedural risks, myocardial biopsies were not performed in the five cases described above.

### 3.2 Differential diagnosis of ICIM

ICIM requires differentiation from other conditions, including acute coronary syndrome (ACS), pulmonary embolism, and viral myocarditis. This differentiation is particularly critical and challenging, especially in patients with coronary artery disease (CAD) risk factors or a prior history of CAD. Even when imaging reveals coronary artery stenosis, ICIM cannot be easily ruled out.For instanc, Cases 1 and 3 had underlying conditions such as hypertension or diabetes mellitus, and both presented with elevated cTnT and coronary artery stenosis—findings that made differentiation from CAD difficult. Yet, they showed no significant improvement with antiplatelet therapy, while immunosuppressive treatment led to marked clinical and biochemical improvement, ultimately confirming the diagnosis of ICIM. According to an international registry report, among 261 patients with clinically suspected ICIM who underwent coronary angiography, 59 (22.6%) were found to have coronary heart disease. Of these, 19 (32.2%) underwent coronary revascularization (22). The interaction of pathological mechanisms suggests that ICIs may significantly increase the risk of ACS by promoting the atherosclerotic process or inducing plaque instability (23). This interaction creates a “double-hit” scenario, further complicating the differential diagnosis.

### 3.3 Treatment of ICIM

#### 3.3.1 Use of glucocorticoids

In the treatment of ICIM, discontinuing ICIs is the initial step. Subsequently, based on the severity of the disease, glucocorticoids are selected for use, and decisions are made on regarding the combination with immunosuppressive agents. Due to the lack of high-quality clinical evidence, there is no unified guideline for the optimal dose of glucocorticoids. The 2022 NCCN guidelines recommend that for patients diagnosed with ICIM, methylprednisolone 1 g/d should be administered immediately for pulse therapy, followed by oral administration after 3–5 consecutive days (24). However, this guideline does not provide a clear graded treatment plan. In contrast, the 2021 ASCO guidelines suggest that patients with grade G1 can be closely observed. For patients with grade ≥ 2, it is recommended to use high-dose glucocorticoids (1–2 mg/kg/d of prednisone, administered orally or intravenously based on the symptoms) as early as possible within 24 h (10). For patients who do not show an immediate response, early use of glucocorticoids at the dose used for preventing heart transplant rejection (methylprednisolone 1 g/d) can be considered (10). Regarding the tapering of glucocorticoids in ICIM, it is currently believed that close follow-up of cTn is necessary (first follow-up 48–72 h after treatment initiation). If cTn levels decrease compared to the baseline and symptoms improve, glucocorticoids can be gradually tapered. Initially, all patients in this study were treated with the regimen of “ICI discontinuation combined with glucocorticoid administration”. With reference to the aforementioned guidelines, the dosage and tapering schedule of glucocorticoids were individually determined, but only one patient was sensitive to glucocorticoids (Table 3).

**Table 3:** Treatment regimens and outcomes of 5 patients.

Treatment/outcome	Case 1	Case 2	Case 3	Case 4	Case 5
Maximum daily dose of methylprednisolone	1 mg/kg	500 mg	2 mg/kg	500 mg	4 mg/kg
Second-line immunosuppressant	Tofacitinib (5 mg BID)	Mycophenolate mofetil (0.75 g BID)	None	Tofacitinib (11 mg/day) + mycophenolate mofetil (0.75 g BID)	None
Total dose of IVIG	None	None	60g	120g	110g
Prophylactic medication	None	TMP-SMX (160/800 mg/day)	None	None	None
Complication	None	None	None	PCP	None
Tumor control status	PR	pCR	PR	SD	Not evaluable (no pre-treatment imaging)
Final outcome	Improved	Improved	Improved	Improved	Death

TMP-SMX, trimethoprim-sulfamethoxazole; PCP, pneumocystis jirovecii pneumonia; PR, partial response; pCR, pathological complete response; SD, stable disease.3. 5 impact of ICIM on tumor prognosis.

#### 3.3.2 Exceptionally high corticosteroid resistance rate in NSCLC-associated ICIM

A striking finding of our study is the 80% glucocorticoid resistance rate (4 out of 5 patients), which is substantially higher than the 50% resistance rate reported in previous large-scale retrospective studies (8, 25). All four glucocorticoid-resistant patients initially received standard-dose glucocorticoids (1–2 mg/kg/day methylprednisolone); however, their cTnT levels failed to decrease by ≥50% within 3–7 days, and in two cases (Cases 1 and 4), cTnT even continued to rise—consistent with the definition of glucocorticoid-resistant ICIM (srICIM) (8). This high resistance rate in our NSCLC cohort warrants attention, as most prior data on srICIM were derived from mixed cancer types (26). Collectively, these findings may remind us of the underappreciated severity and treatment resistance of ICIM in NSCLC. They could challenge the management paradigm largely derived from broader oncology cohorts, underscoring the need for increased NSCLC-specific vigilance. Consequently, clinicians may benefit from proactively monitoring for steroid resistance in this subgroup, and lowering the threshold for prompt escalation to second-line immunosuppression to reduce the high risk of life-threatening complications.

#### 3.3.3 PCP under combined immunosuppression

Our case data underscore the critical role of prophylaxis during ICIM treatment. Case 2, who received combined high-dose glucocorticoids, one immunosuppressant and TMP-SMX prophylaxis as recommended, remained free of PCP. In contrast, Case 4, treated with high-dose glucocorticoids and two immunosuppressants but without prophylaxis, subsequently developed PCP. Although the patient recovered following anti-PCP therapy, cTnT levels rebounded, after which mycophenolate mofetil was safely reintroduced at a reduced dose. These observations highlight key clinical implications: in srICIM patients receiving combined immunosuppression, clinicians should closely monitor respiratory symptoms, implement tailored prophylaxis, and promptly differentiate between PCP co-infection, ICIM exacerbation, and tumor progression. Once PCP is confirmed, coordinated management of both conditions is essential to avoid compromising the outcome.

#### 3.3.4 Efficacy of tofacitinib and mycophenolate mofetil as second-line therapies for srICIM

Against this high glucocorticoid resistance background, our case series provides real-world evidence for the efficacy of tofacitinib and mycophenolate mofetil as second-line agents—a critical gap in current guidelines, which lack specific recommendations for srICIM. Case 1, a 64-year-old male with probable ICIM, showed no response to 1 mg/kg/day methylprednisolone; after adding tofacitinib (5 mg twice daily), his cTnT levels progressively normalized, and tumor control (partial response, PR) was maintained. Case 4, a 67-year-old female with possible ICIM and immune-mediated myositis, failed both standard glucocorticoids and methylprednisolone pulse therapy (500 mg/day for 3 days); the combination of tofacitinib (11 mg once daily) and mycophenolate mofetil (0.75 g twice daily) led to rapid resolution of ptosis and a gradual decrease in cTnT. These outcomes align with the mechanistic rationale of tofacitinib (a JAK1/JAK2 inhibitor) in inhibiting excessive T-cell activation via the JAK-STAT pathway (27) and mycophenolate mofetil (a purine analog) in inhibiting lymphocyte proliferation (28). Notably, our data extend prior small case series (26, 29) by demonstrating efficacy in NSCLC-specific ICIM and supporting the use of combination second-line therapy for patients unresponsive to single-agent immunosuppression.

In the current treatment strategies, second-line therapies for srICIM include, in addition to the two aforementioned agents, other immunosuppressants such as tacrolimus, biological agents including infliximab, tocilizumab, and alemtuzumab, as well as antithymocyte globulin, abatacept, and IVIG (10, 25, 30–33). Another potential treatment is plasma exchange, which removes antigens and antibodies from the plasma and inhibits the excessive immune response. A retrospective analysis suggests that combining plasma exchange with glucocorticoid therapy may be more effective than glucocorticoids alone in patients with ICI-induced myocarditis (34).

### 3.4 Fulminant myocarditis associated with ICIs

Fulminant myocarditis is a severe and distinct form of acute myocarditis. However, early clinical identification remains challenging. Once the myocardial inflammatory response occurs, the disease progresses rapidly, often leading to hemodynamic instability, severe arrhythmias, and multi-organ damage (e.g., liver and kidney injury) (35). Although epidemiological data specific to ICI-associated fulminant myocarditis are currently limited, a recent observational study of 48 patients with ICIM reported a mortality rate as high as 87.5% in fulminant cases (36).

Traditional treatment for this condition relies heavily on clinical experience and includes hemodynamic support, high-dose glucocorticoid pulse therapy, and IVIG administration (35). However, in recent years, there have been some advancements in the treatment strategies for ICI-related fulminant myocarditis. The key breakthrough lies in the use of a comprehensive treatment plan that combines multi-target immunosuppression with hemodynamic support. A study published by a team from Sorbonne University in France in 2023 demonstrated that early administration of abatacept (a CTLA-4 agonist) combined with ruxolitinib (a JAK1/JAK2 inhibitor) can reduce the mortality rate of fulminant myocarditis from 60% with traditional treatment to just 3% (37). In cases where patients are critically ill and experiencing cardiogenic shock, veno-arterial extracorporeal membrane oxygenation (VA-ECMO) can be used as a salvage therapy, providing a crucial time window for the effectiveness of immunosuppressive treatment (38, 39). It is important to note that a multidisciplinary collaborative approach to diagnosis and treatment, involving disciplines such as cardiology, respiratory medicine, critical care medicine, oncology, and rheumatology, can greatly improve the success rate of rescuing patients.

In our study, one patient (Case 5) with grade G4 fulminant myocarditis died despite receiving multidisciplinary treatment, which is consistent with the high fatality rate of ICIM reported in previous studies (5, 7). However, a critical limitation of our mortality attribution in this case warrants explicit acknowledgment: the patient concurrently presented with severe pneumonia, septic shock, and multi-organ failure, which may have collectively contributed to mortality—potentially overestimating the sole role of fulminant ICIM. This death may also have been influenced by the delayed initiation of glucocorticoid therapy.

### 3.5 Impact of ICIM on tumor prognosis

The relationship between irAEs and tumor prognosis during ICI treatment is complex and exhibits a positive “toxicity-efficacy” association. Clinical studies have demonstrated that NSCLC patients experiencing irAEs have significantly better median overall survival (OS) and median progression-free survival (PFS) compared with those without such irAEs (39–41). This survival benefit is particularly evident in patients with low-grade (G1-2) toxicity, while high-grade (G3-4) toxicity may impair therapeutic efficacy due to treatment interruption or the need for immunosuppression (39–41). A retrospective analysis of 66 lung cancer patients with ICI-related cardiac toxicity revealed that the median PFS for NSCLC patients was 24.4 months (42). These results appear superior to previous clinical trial data from immunotherapy trials, such as the combination of pembrolizumab and chemotherapy in advanced lung squamous cell carcinoma, which had a median PFS of only 8 months (21), and the combination of nivolumab and chemotherapy in advanced non-squamous NSCLC, which had a median PFS of only 9 months (43).

Among the five cases reported in this study, one critically ill patient died despite treatment. However, owing to the absence of pre-treatment chest imaging data provided by the patient's family, it was not possible to evaluate the anti-tumor efficacy. The other four patients discontinued ICI treatment after being diagnosed with ICIM. During the 5-week to 5-month interrupted anti-tumor therapy, the tumors did not show significant progression (two cases with PR, one case with SD, and one case with pCR. This seemingly contradictory phenomenon may be attributed to sustained effects of immune memory, which can maintain anti-tumor activity even when treatment is interrupted. Future research should focus on developing multi-omics predictive markers, finding a balance between toxicity control and efficacy maintenance, and developing novel strategies for precision immunotherapy.

## 4 Conclusion

This retrospective study analyzed five cases of NSCLC patients who developed ICIM during ICI therapy. The results indicate that although ICIM is a relatively rare complication in NSCLC treatment, it is a life-threatening adverse event that necessitates timely identification and prompt intervention. The diagnosis of ICIM requires integration of patients’ clinical manifestations and multimodal diagnostic assessments. Treatment involves discontinuing ICIs and initiation of glucocorticoid therapy; however, the optimal glucocorticoid dosage and tapering strategy remain undefined. Several second-line treatment options are available for patients with glucocorticoid-resistant ICIM, but no consensus-based specific recommendations have been formulated. It is recommended to formulate individualized treatment strategies via MDT. Fulminant myocarditis, a severe form of ICIM, has an extremely high fatality rate, and its management remains largely empirical. However, the cases in this study demonstrate that ICIM does not compromise short-term tumor control outcomes. Future research should focus on undertaking additional prospective studies to refine diagnostic criteria, optimize treatment strategies, and construct validated risk prediction models in order to improve the management of ICIM and uphold the safety and efficacy of immunotherapy in NSCLC patients.

## Data Availability

The original contributions presented in the study are included in the article/Supplementary Material, further inquiries can be directed to the corresponding author.
